# Supplementary material for: Impact of Face-to-Face Teaching in Addition to Electronic Learning on Personal Protective Equipment Doffing Proficiency in Student Paramedics: Randomized Controlled Trial
Source: Int J Environ Res Public Health. 2022 Mar 5;19(5):3077. doi: 10.3390/ijerph19053077 (PMC8910255; doi:10.3390/ijerph19053077)
Supplement: Supplementary file 1 [file ijerph-19-03077-s001.zip › Supplementary File S1. PPE doffing procedure for instructors.pdf]

This is a Supplementary File to a full manuscript published in *International Journal of Environmental Research and Public Health*. For full copyright and citation information see article.

Table S1. PPE doffing procedure for instructors

French original version	English translated version
**Procédure retrait EPI – À destination des formateurs·rices**	**PPE doffing procedure – For instructors**
1. Rester dans la zone contaminée	1. Stay in contaminated zone
2. Retrait et élimination non contaminant des gants (poubelle)	2. Non-contaminating removal and disposal of gloves (trash can)
3. Friction hydroalcoolique des mains	3. Hydroalcoholic hand rubbing
4. Ouvrir la combinaison	4. Open the coverall
5. Friction hydroalcoolique des mains	5. Hydroalcoholic hand rubbing
6. Enlever la capuche	6. Take off the hood
7. Ôter la combinaison en l'enroulant depuis l'intérieur, du haut jusqu'aux chevilles	7. Take off the coverall by rolling it up from the inside, from the top to the ankles
8. Jeter la combinaison dans une poubelle fermée	8. Dispose the coverall in a closed trash can.
9. Passer en zone « non-contaminée »	9. Go in non-contaminated zone
10. Friction hydroalcoolique des mains	10. Hydroalcoholic hand rubbing
11. Retrait non contaminant des protections oculaires	11. Non-contaminating removal of eye protection
12. Placer les lunettes dans un sachet, sans toucher celui-ci, pour désinfection ultérieure	12. Place the glasses in a bag, without touching it, for subsequent disinfection
13. Friction hydroalcoolique des mains	13. Hydroalcoholic hand rubbing
