# Supplementary material for: Impact of Face-to-Face Teaching in Addition to Electronic Learning on Personal Protective Equipment Doffing Proficiency in Student Paramedics: Randomized Controlled Trial
Source: Int J Environ Res Public Health. 2022 Mar 5;19(5):3077. doi: 10.3390/ijerph19053077 (PMC8910255; doi:10.3390/ijerph19053077)
Supplement: Supplementary file 1 [file ijerph-19-03077-s001.zip › Supplementary File S2.pdf]

This is a Supplementary File to a full manuscript published in International Journal of Environmental Research and Public Health. For full copyright and citation information see article.

Table S2. Assessment grid

French original version	English translated version
**Numéro de la session (1 ou 2)**	**Session number (1 or 2)**
**Identifiant du participant**	**Participant identifier**
**Durée de la séquence** *(en secondes ; dès que le premier élément est saisi jusqu'à ce que la procédure soit signalée comme terminée)*	**Sequence duration** *(in seconds; from when the first element is taken until the procedure is completed)*
**<u>Déshabillage</u>**<br>*Les étapes suivantes doivent être suivies dans l'ordre. L'absence d'une des actions citées ci-après <u>OU</u> le non-respect de l'ordre de la séquence représente une contamination (échec de la procédure)*	**<u>Doffing</u>**<br>*The following steps should be taken in order. The absence of one of the actions listed below <u>OR</u> the non-compliance with the order of the sequence represents contamination (failure of the procedure*)
**En zone contaminée**	**In contaminated zone**
□ Retrait non contaminant des gants	□ Non-contaminating glove removal
□ Friction hydroalcoolique des mains	□ Hydroalcoholic hand rubbing
□ Ouverture de la combinaison	□ Coverall opening
□ Friction hydroalcoolique des mains	□ Hydroalcoholic hand rubbing
□ Retrait de la combinaison sans contact avec l'extérieur de celle-ci (prêter particulièrement attention à la capuche) ; la jeter dans une poubelle fermée	□ Coverall removal without outside contact of it (pay particular attention to the hood); throw it in a closed trash can
**En zone non-contaminée**	**In non-contaminated zone**
□ Friction hydroalcoolique des mains	□ Hydroalcoholic hand rubbing
□ Retrait des lunettes de protection ; les placer dans un sachet (sans le toucher) pour une désinfection ultérieure	□ Protective glasses removal; placed them in a bag (without touching it) for subsequent disinfection
□ Friction hydroalcoolique des mains	□ Hydroalcoholic hand rubbing
